# Thoughts on “Estimation of radiation exposure of children undergoing superselective, intra-arterial chemotherapy for retinoblastoma treatment: Assessment of Local Diagnostic Reference Levels as a function of age, sex and interventional success”

**DOI:** 10.1007/s00234-020-02594-7

**Published:** 2020-11-03

**Authors:** Marcel Opitz, Axel Wetter, Nika Guberina

**Affiliations:** 1 Institute of Diagnostic and Interventional Radiology and Neuroradiology, University Hospital Essen, Hufelandstraße 55, 45147 Essen, Germany; 2 Clinic of Radiotherapy, University Hospital Essen, West German Cancer Center, Essen, Germany

Dear Editor,

We would like to thank the authors of the “Letter to the Editor” concerning our manuscript “Estimation of radiation exposure of children undergoing superselective intra-arterial chemotherapy for retinoblastoma treatment: Assessment of Local Diagnostic Reference Levels as a function of age, sex and interventional success” [1] for their feedback in this important and exciting field of research.

The principle aim of our study was to establish pediatric diagnostic reference levels (DRLs) for intra-arterial chemotherapy (IAC) procedures. We absolutely agree with the authors that pediatric DRLs for IAC procedures will increase dose awareness, and in the long term, optimize the modification of equipment, technique, and imaging parameters. Also, the International Commission on Radiological Protection (ICRP) and the European Guidelines on Diagnostic Reference Levels for Paediatric Imaging are proclaiming the necessity for DRLs for pediatric patients [2, 3], so work like ours may probably contribute to the establishment of DRLs in IAC.

In the European Guidelines on Diagnostic Reference Levels for Paediatric Imaging in cranial examinations, age is recommended as the grouping parameter [3]. For this reason, we determined the DAP and fluoroscopy time values according to the recommended age groups. Since no alternative routes through the external carotid artery were probed for drug administration in our patients, it did not make sense for us to divide the procedure into distinct phases. This deliberate approach pursued in our center is described in detail by Stenzel et al. [4].

We read with great interest the authors’ work [5, 6]. In contrast to our colleagues, we did not determine absolute dose values with anthropomorphic phantoms, but concentrated ourselves on the DRLs which are based on the DRLs for other interventional radiological procedures published by the Federal Office for Radiation Protection in Germany [7]. Contrary to phantom measurements, DRLs are a practical and feasible way of radiation exposure comparison of different devices at different sites in the clinical routine.

In conclusion, we can only welcome a lively discussion on this topic highlighting the necessity of radiation dose awareness, hopefully leading to further dose optimization.
